# Empowering Patients With a Shared Communication Tool: A Patient-Oriented Multimethods Pilot Study

**DOI:** 10.1177/23743735231160421

**Published:** 2023-03-09

**Authors:** Sharla King, Melanie Garrison, Manon Fraser, Michelle Wiley, Heidi Sharek, Sharon Gaine, Wanda Kosteroski

**Affiliations:** 1 Faculty of Education, 3158 University of Alberta, Edmonton, Alberta, Canada; 2 3146 Alberta Health Services, Edmonton, Alberta, Canada; 3 Family Advisor, 60351 Glenrose Rehabilitation Hospital, Edmonton, Alberta, Canada; 4 Department of Audiology, Glenrose Rehabilitation Hospital, Edmonton, Alberta, Canada; 5 Department of Psychology, Glenrose Rehabilitation Hospital, Edmonton, Alberta, Canada; 6 Department of Social Work, Glenrose Rehabilitation Hospital, Edmonton, Alberta, Canada

**Keywords:** shared decision-making, Plan-Do-Study-Act cycle, empowerment, collaborative culture

## Abstract

Not all patients feel empowered to take on the expanding role as active members in their healthcare journey. Healthcare services must shift attention to supporting patients and families in this emerging role. This support includes providing communication tools designed for patients and families to empower them to speak up. Two Plan-Do-Study-Act (PDSA) cycles were conducted to test a communication tool, the *Jargon Alert!/WAIT* card, with patients/families and providers in a Canadian rehabilitation hospital. After the first PDSA cycle, feedback from patients/families (n  =  24), and providers (n  =  4), informed modifications. The new *Question Alert!* card was retested in the same clinics. Patients/families (n  =  13) reported the new card was a valuable tool enabling them to ask questions, although not all patients or family members expressed the need to use the card. The participating providers (n  =  4) thought the *Question Alert!* card was helpful for quieter patients or family members who normally shy away from asking questions. The shared communication tool designed with patients improved the patient-centered experience and empowered patients/families to be more involved in their care.

## Introduction

Enacting person-centered care (PCC) means the role of the patient shifts to include them as active members and shared decision-makers in their care (1, 2). However, not all patients feel confident or competent to speak up to their providers. Patients don’t want to be seen as a trouble-maker to their providers by asking questions (2, 3), or they may not know what to say, especially if concerned about mismatched care goals, or confusing/conflicting information (3). Even well-educated patients are hesitant to speak up to their physicians to avoid the “difficult” patient label (4). Patients’ understanding of shared decision-making (SDM) may vary depending on their race, ethnicity, or educational level (5, 6). Additionally, not all patients are ready to assume an active role in their healthcare, particularly when newly diagnosed; their level of involvement may evolve over time (7). As such, healthcare services and systems must shift attention to supporting patients and families in this emerging role (8).

Standardized, structured approaches and tools to support effective plain language communication optimize shared understanding, SDM, and patient- and family-centered care (9). Health care professionals receive communication training (10), primarily designed to optimize communication between providers and patients/families (11-13). Unfortunately, patients and family members generally do not have awareness or training with these same tools, nor are these tools designed to be shared with patients and families. Patient decision aids (PtDAs) are resources designed for patients to outline the options for decisions in areas such as surgery, screening, and medication treatments (14). Patient decision aids improve people's knowledge of their options so they feel better informed (14), yet preparing patients for the SDM encounter goes beyond patients possessing knowledge about their care (15). Foundational to implementing SDM is strong, transparent, and comprehensible communication between providers and patients (16).

The purpose of this project was to rapidly evaluate a communication tool for patients/families, *Jargon Alert!/WAIT* card, for use with their providers at a rehabilitation hospital. The goal of the card was 2-fold: First, it was to empower patients/families to engage in plain language communication with their rehabilitation team. Second, it was to remind providers to slow down, allow time for questions and provide jargon-free information to patients and families. This easy-to-use card may promote a shared understanding between patients/families, and providers and individual awareness, attitudes, and behaviors to enhance open communication, collaboration, and PCC.

### Evolution of the *Jargon Alert!/WAIT* Card

A communication tool (business card size to clip to a lanyard) designed to support provider-provider jargon-free communication was redesigned to share with health professional students in an interprofessional course. The cards had *Jargon Alert!* on one side, and the acronym *WAIT (Why Am I Talking)* on the other side as a reminder that active listening contributes to effective communication. The *Jargon Alert!/WAIT* card was shared at academic and clinical conferences. At one of these presentations, patients and family members in the audience expressed the need for this card to empower them to speak up during appointments. The *Jargon Alert!/WAIT* card was then introduced at a workshop hosted by the Self-Management Support Committee at a Canadian rehabilitation hospital and led by 2 university researchers. The workshop was attended by providers and patients/family advisors from the hospital. The positive reaction to the card at the workshop sparked the formation of a study team with self-identified staff, providers, and family advisors and university researchers to pilot-test the card as a patient- and family-communication tool in select clinics at the hospital.

## Methods

The study team followed a participatory action research approach (17) to guide the development and implementation of this project. Two iterative Plan-Do-Study-Act (PDSA) cycles were conducted to pilot test the implementation and design of the existing *Jargon Alert!/WAIT* card with patients/families and providers. PDSA cycle, commonly used in healthcare quality improvement, was the selected method because of its methodological features of iterative cycles, small-scale testing, and continuous data collection (18). PDSA cycles are a pragmatic approach to rapidly test and assess interventions in complex social environments, allowing researchers to flexibly adapt the intervention according to feedback from each cycle, thus minimizing risks to patients and the organization and providing evidence for conducting a larger scale research study (18).

Evaluation evidence was collected to pilot the utility of the tool to support patients/families in a clinical setting and to explore improvements to the tool. Four providers from the study team (1 audiologist [1:1 setting], 1 social worker from the Autism clinic—program for complex patients group setting, 2 psychologists from specialized rehabilitation outpatient program [1:1 and group settings]) volunteered to introduce the tool to their patients. A provider implementation package was created including a visual aid and a user guide to support consistent and effective explanations about the purpose and use of the card. The Glenrose Rehabilitation Hospital is the largest freestanding, comprehensive tertiary rehabilitation hospital in Canada committed to ensuring patients and families are at the center of all healthcare activities, decisions, and teams, providing an ideal clinical environment to pilot test the tool. Ethical approval for the study was obtained through the Human Research Ethics Board at the University of Alberta and the Glenrose Research Ethics with Alberta Health Services. (Study ID# Pro00089439).

### Procedures

The *Jargon Alert!/WAIT* card was piloted with patients and family members in group and 1:1 settings over a 4-month period. All patients/families who visited the participating providers for an appointment were invited to use the card during the interaction. After their appointments, the patients and/or family members were asked to complete a paper-based survey. The providers who introduced the card to their patients/families also completed an online survey following the first round of testing.

Based on the patient/family feedback (n  =  24), the overall design of the tool was maintained. The patients/families identified that not all communication issues were a “jargon thing,” and there was confusion about the purpose of the *WAIT* side of the card. Due to this feedback, the card was revised and transitioned to the *Question Alert!* Card. Phrases taken from qualitative feedback from patients and families were incorporated into the card with one side indicating “I have a question” with a question mark symbol and the other side of the card providing prompts for the patients/families to assist with starting the conversation. The *Question Alert!* card was evaluated in PDSA Round 2 using the same surveys from Round 1. Round 2 was conducted with the same providers in group and 1:1 settings.

### Surveys

The study team co-developed the surveys (see Supplemental Material). Although the surveys were not piloted, the co-development process ensured meaningful items were asked in a short, easy-to-respond to format. The patient/family survey asked about the introduction of the card, if they used it, how the provider responded, and if they would use it again. Responses were either Yes or No. Open-response questions asked how they felt about the card and for other suggestions. The survey for the providers used a 7-point Likert scale with anchors of strongly disagree to strongly agree to better capture the providers’ true evaluation of the tool. Items asked about the impact of introducing the card, if the card supported PCC approach, and if they would use the card again. Open-response questions asked about the experience of using the card and if changes to the card were necessary. Survey questions were analyzed by descriptive statistics. Responses to the open-ended questions were reported.

## Results

### PDSA Round 1: *Jargon Alert!/WAIT*

The majority of patient/family respondents (79%) stated the design of the card was helpful, they were comfortable using the card (79%) and they would use the card in the future (88%), even though only 50% used the card (Table 1). Respondents thought the card was helpful and was “easier than raising hand” in a group setting. However, several respondents commented on the word “jargon” and were confused with the term *WAIT* (Table 2).

**Table 1.** Patients’/Family Members’ and Providers’ Responses to Closed-Response Survey Questions About *Jargon Alert/WAIT* Card and *Question Alert!* Card (Round 1 and 2).


	Group Setting (n = 15) % (n)					1:1 Interaction (n = 9) % (n)					Total (n = 24) % (n)			
**Round 1: *Jargon Alert/WAIT* Card**	Yes		No		NR	Yes	No			NR	Yes	No	NR	
Did you use the card?	67 (10)		33 (5)		0	22 (2)	78 (7)			0	50 (12)	50 (12)	0	
Was the healthcare provider clear in how they asked you to use the card?	93 (14)		0		7 (1)	100 (9)	0			0	96 (23)	0	4 (1)	
Did the healthcare provider answer in the way you thought they would when you used the card?	73 (11)		0		27 (4)	100 (9)	0			0	83 (20)	0	17 (4)	
Did the healthcare provider slow down or stop when you used the card?	73 (11)		0		27 (4)	44 (4)	0			56 (5)	63 (15)	0	38 (9)	
Were you comfortable using the card?	87 (13)		0		13 (2)	67 (6)	22 (2)			11 (1)	79 (19)	8 (2)	13 (3)	
Would you use the card at a future session?	87 (13)		0		13 (2)	89 (8)	11 (1)			0	88 (21)	4 (1)	8 (2)	
Is the design of the card helpful?	80 (12)		0		20 (3)	78 (7)	0			22 (2)	79 (19)	0	21 (5)	

	Group Setting (n = 6) % (n)					1:1 Interaction (n = 7) % (n)					Total (n = 13) % (n)			
**Round 2: Question Alert! Card**	Yes	No		NR	N/A	Yes	No		NR	N/A	Yes	No	NR	N/A
Did you use the card?	100 (6)	0		0	0	14 (1)	86 (6)		0	0	54 (7)	46 (6)	0	0
Did having the card offered/ explained to you influence how you interacted during the session?^a^	100 (6)	0		0	0	86 (6)	14 (1)		0	0	92 (12)	8 (1)	0	0
Was the Health care provider clear in how they asked you to use the card?	100 (6)	0		0	0	100 (7)	0		0	0	100 (13)	0	0	0
Did the Health care provider answer your question clearly?	100 (6)	0		0	0	57 (4)	0		14 (1)	29 (2)	77 (10)	0	8 (1)	15 (2)
Did the health care provider slow down or stop why you used the card?	100 (6)	0		0	0	43 (3)	0		14 (1)	43 (3)	69 (9)	0	8 (1)	23 (3)
Were you comfortable using the card?	100 (6)	0		0	0	57 (4)	0		14 (1)	29 (2)	77 (10)	0	8 (1)	15 (2)
Will you keep the card to use it elsewhere?	67 (4)	33 (2)		33 (2)	0	100 (7)	0		0	0	85 (11)	15 (2)	0	0
Is the design of the card helpful?	100 (6)	0		0	0	100 (7)	0		0	0	100 (13)	0	0	0

**Providers Responses Rounds 1 & 2**	Round 1 n = 4							Round 2 n = 4						
The introduction of the card fit into my regular interactions with the client/patient/family.	25% Strongly Agree 50% Agree 25% Somewhat Agree							25% Strongly Agree 75% Agree						
The card served as a reminder to me to slow down or seek clarification with the patients/clients/families.	25% Strongly Agree 50% Somewhat Agree 25% Neither Agree nor Disagree							25% Strongly Agree 25% Agree 25% Somewhat Agree 25% Neither Agree nor Disagree						
The card supported the patient/client/family-centered approach or philosophy.	25% Strongly Agree 75% Agree							25% Strongly Agree 50% Agree 25% Somewhat Agree						
The card changed my session with the client/patient/family.	25% Somewhat Agree 25% Neither Agree nor Disagree 25% Disagree 25% Strongly Disagree							25% Somewhat Agree 75% Neither Agree nor Disagree						
The card was easy to use.	25% Strongly Agree 25% Agree 50% Neither Agree nor Disagree							50% Strongly Agree 25% Agree 25% Somewhat Agree						
The card had an impact on the interaction.	25% Agree 50% Somewhat Agree 25% Neither Agree nor Disagree							75% Somewhat Agree 25% Neither Agree nor Disagree						
There was a benefit to the client/patient/family in using the card.	25% Strongly Agree 25% Agree 50% Neither Agree nor Disagree							25% Strongly Agree 50% Somewhat Agree 25% Neither Agree nor Disagree						
There was a benefit to me as a clinician in using the card.	25% Strongly Agree 50% Somewhat Agree 25% Neither Agree nor Disagree							25% Strongly Agree 25% Agree 25% Somewhat Agree 25% Neither Agree nor Disagree						
I would use the card again.	25% Strongly Agree 75% Agree							25% Strongly Agree 50% Agree 25% Somewhat Agree						

Abbreviation: NR, no response.

^a^ This question was added to Round 2 based on discussions with the providers on the study team.

**Table 2.** Responses From Patients’/Family Members’ and Providers’ to Open-Ended Questions for Rounds 1 and 2.

**Patients/Family Members**	
*Jargon Alert!/WAIT* Round 1	Question Alert! Round 2
How did you feel using the card	
9 respondents commented I liked it but sometimes it was forgotten by others. I would try to grab it to show and wait. I used it over the course of 2 days. Good. Felt easier than raising hand. Very helpful Very helpful and straightforward More specific, very helpful It's a very good idea It is a really good idea Easy Did not need to use it	7 respondents commented It's a good idea for getting answers repeated. Comfortable Great! it's empowering! I think it helps the health provider realize you want clearer/better info, etc! Not as confident as I should have. It was easy and find. Fine – don’t have a problem interrupting. I felt comfortable using this tool to ask a question as it was an established tool and I didn't feel like I was interrupting.
Any other thoughts or suggestions?	
11 respondents commented Very good idea. I am not sure the “Jargon” term is the sort to use. Many questions raised were not the Jargon sort. Just wanted questions answered. It would be helpful to have “I have a question/comment. I don't understand why WAIT means “Why am I Talking”. Otherwise it is a great idea! Sometimes I used the WAIT and Jargon side wrong. I think it could be “WAIT” I have a question instead of slow down and “Jargon” is I don’t understand and repeat what you said or explain in better language. I didn’t use the card however had I needed to I would have. I think it's an excellent tool for workshops and seminars. WAIT card should be used for questions. Confusing for *Jargon Alert* questions. The “Why am I talking” doesn’t make sense to me. How about a simple “Please slow down”? I am not the type to use this card. I can just ask myself.	4 respondents commented I am a very open and strong personality. I have no problems to stop the conversation and ask. Wonderful idea (smiley face inserted). Just didn't need it today. Unless other facilities understand the card, I may feel silly using the card. One for a child!
**Provider Responses**	
*Jargon Alert!/WAIT* Round 1	Question Alert! Round 2
The card, while helpful, did slow us down. In this scenario the card was applied in a group setting - so likely benefited the individual but may have hindered others as we were not able to cover all the content. Explaining the WAIT side better, confusing to patient. I think the cards are a wonderful addition to the workshop/group.	I liked the cards for the quieter participants that sometimes get overwhelmed when they have a question but feel shy to ask. Many different people used the card, no one raised their hands or spoke out/interrupted. I am always open to questions throughout my sessions so it was about the same regardless of whether they used the card or just put up their hand to ask a question. With the number of questions, comments, discussion, I rushed through the last part of my presentation. I enjoy using the cards and having the families go home with them to use them in other health environments

All providers agreed that it could be accommodated into regular patient interactions (Table 1), despite the feedback highlighting the extra time needed to explain the card (Table 2). Half of the providers felt introducing the card did not change their session. Half of the providers felt there was a benefit to the patients/families in using the card. Providers saw the card as a tool to support PCC (100% strongly agree/agree), and all responded they would use the card again. Providers also noted the *WAIT* side of the card did not easily translate during the interactions with the patients/family members.

### PDSA Round 2: Question Alert!

Thirteen patients/family members (6 from group and 7 from 1:1 settings) participated in Round 2. Seven participants (6 in group setting and 1 in the 1:1 setting) used the card, yet 92% of the respondents felt the inclusion of the card influenced how they interacted during the session. All of the participants stated the provider clearly explained how to use the card. The majority of participants (85%) indicated they would keep the card to use elsewhere, and all participants (100%) felt the design of the card was helpful.

Open-response comments from patients/family members (Table 2) indicated not all participants felt they needed to use the card as they are confident to speak up, yet they still thought it was helpful to have the card. One participant voiced concern that a lack of consistency of the card across healthcare contexts may decrease comfort in using the card. No additional suggestions about the design of the card arose during Round 2.

Again, all providers agreed the introduction of the card fit into their regular interactions. However, one provider did express they had to rush their session to finish because of the card (Table 2). The majority of the providers (75%) felt the card supported PCC, it served as a reminder to slow down and it benefited patients/families, particularly the quieter participants. All agreed they would use the card again.

## Discussion

Conducting 2 PDSA cycles quickly provided the study team with information about the utility of the *Jargon Alert!/WAIT* card with patients/families and providers in a clinical setting. The feedback from Round 1 confirmed that patients/families would use the card in the future. Prioritizing patient/family feedback for card design transitioned the card to the *Question Alert!* card and ensured patient/family-centered engagement and resources. Although explaining the card takes time, providers reported integrating the card was feasible and they would continue to use it in the future. In Round 2, patients/families indicated the card impacted the environment including empowering them to ask questions, regardless if respondents used the card during the session. The providers thought that the *Question Alert!* card was helpful as a reminder to slow down and support typically quieter patients/family members, yet inclusion could impact the length of interaction.

The power of the *Question Alert!* card may not only be in utilizing the card as anticipated but also in the awareness of the card for both patients/families and providers. Explaining to patients/families the purpose of the card and encouraging them to use it, gives “permission” to the patient and family member to speak up, ask questions, and reinforce their voice matters. Although the card may not be necessary for everyone, it minimizes assumptions made by providers that all patients/families are able to communicate their preferences during an appointment and have the desire to actively participate in their care (7, 19). Providing this simple card reinforces the idea that the patient has “permission” to ask questions. Inclusion of the card may externalize professional and organizational PCC rhetoric and address assumptions that the patient/family role within PCC is well understood by patients/families and providers/staff (19). Creating a culture for SDM with patients/families by including the card as part of an SDM implementation program may better support patients/families to embrace their role as a team member. As identified in the literature (8, 15), this pilot study supports the provision of standardized, structured approaches and tools to ensure effective plain-language communication. Aligning with PtDA research (14), the Question Alert! Card supports patients/families engaging in transparent and clear communication with their providers ideally improving SDM. The increased time needed to explain the card and accommodate questions and discussion may prove to be a hindrance as healthcare systems prioritizing efficiencies may overshadow the effectiveness of care interactions. The realities of implementing PCC may not align with clinical resource capacities (2).

## Limitations

There are 3 key limitations. First, only members of the study team piloted the cards with patients/families, thus the provider results may be due to their involvement in the study and their support of SDM. Second, we did not collect detailed demographic information about the participants. As this was a PDSA intended to quickly determine the acceptability and usability of the card in a clinical setting before scaling up to a larger implementation, we wanted to minimize the burden on patients/families and providers. Finally, the rehabilitation hospital where the pilot was conducted has a strong PCC philosophy, thus this context may not be representative of nor generalizable to other care environments. This pilot study was designed to quickly gather immediate feedback on the utility of the card in a clinical environment to support patient/family member communication with their provider. One study respondent raised the issue of introducing the card in a clinical setting where the purpose of the card was unfamiliar to the providers. The introduction of the card to providers outside of the study team is a critical point to ensure patients/family members feel empowered to use the card when necessary.

## Conclusion

The PDSA cycles demonstrated the benefit of the card for patients/families in a clinical context and provided early evidence justifying conducting a larger-scale study to examine whether and how the card supports SDM and patient empowerment. To enact PCC, patients/family must feel empowered to ask questions, seek clarification, and fully engage in SDM. For some, this comes naturally. For others, encouragement or support is needed to empower them to actively participate. Providers may mistakenly assume that patients are aware of their active role and able to communicate their needs. A communication tool for patients and their family members may provide that additional support. Further, a shared communication tool designed with and for patients/families can foster a collaborative culture within the healthcare context and across patients, their families, staff, and providers.

## Supplemental Material

sj-pdf-1-jpx-10.1177_23743735231160421 - Supplemental material for Empowering Patients With a Shared Communication Tool: A Patient-Oriented Multimethods Pilot Study Click here for additional data file. Supplemental material, sj-pdf-1-jpx-10.1177_23743735231160421 for Empowering Patients With a Shared Communication Tool: A Patient-Oriented Multimethods Pilot Study by Sharla King, Melanie Garrison and Manon Fraser, Michelle Wiley, Heidi Sharek, Sharon Gaine, Wanda Kosteroski in Journal of Patient Experience

sj-pdf-2-jpx-10.1177_23743735231160421 - Supplemental material for Empowering Patients With a Shared Communication Tool: A Patient-Oriented Multimethods Pilot Study Click here for additional data file. Supplemental material, sj-pdf-2-jpx-10.1177_23743735231160421 for Empowering Patients With a Shared Communication Tool: A Patient-Oriented Multimethods Pilot Study by Sharla King, Melanie Garrison and Manon Fraser, Michelle Wiley, Heidi Sharek, Sharon Gaine, Wanda Kosteroski in Journal of Patient Experience

sj-pdf-3-jpx-10.1177_23743735231160421 - Supplemental material for Empowering Patients With a Shared Communication Tool: A Patient-Oriented Multimethods Pilot Study Click here for additional data file. Supplemental material, sj-pdf-3-jpx-10.1177_23743735231160421 for Empowering Patients With a Shared Communication Tool: A Patient-Oriented Multimethods Pilot Study by Sharla King, Melanie Garrison and Manon Fraser, Michelle Wiley, Heidi Sharek, Sharon Gaine, Wanda Kosteroski in Journal of Patient Experience

sj-pdf-4-jpx-10.1177_23743735231160421 - Supplemental material for Empowering Patients With a Shared Communication Tool: A Patient-Oriented Multimethods Pilot Study Click here for additional data file. Supplemental material, sj-pdf-4-jpx-10.1177_23743735231160421 for Empowering Patients With a Shared Communication Tool: A Patient-Oriented Multimethods Pilot Study by Sharla King, Melanie Garrison and Manon Fraser, Michelle Wiley, Heidi Sharek, Sharon Gaine, Wanda Kosteroski in Journal of Patient Experience
