# Supplementary material for: Empowering Patients With a Shared Communication Tool: A Patient-Oriented Multimethods Pilot Study
Source: J Patient Exp. 2023 Mar 9;10:23743735231160421. doi: 10.1177/23743735231160421 (PMC10009027; doi:10.1177/23743735231160421)
Supplement: sj-pdf-1-jpx-10.1177_23743735231160421 - Supplemental material for Empowering Patients With a Shared Communication Tool: A Patient-Oriented Multimethods Pilot Study [file sj-pdf-1-jpx-10.1177_23743735231160421.pdf]

Jargon Alert!

UNIVERSITY OF ALBERTA

IPCLU
Interprofessional Clinical Learning Unit

sjk1@ualberta.ca

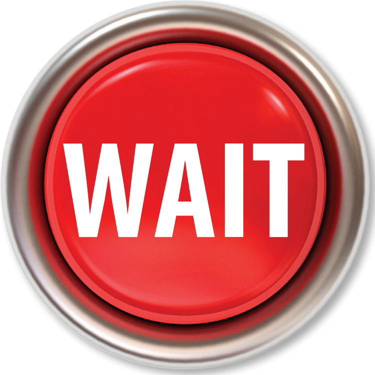

WAIT = **W**hy **A**m **I** **T**alking?
