# Supplementary material for: Empowering Patients With a Shared Communication Tool: A Patient-Oriented Multimethods Pilot Study
Source: J Patient Exp. 2023 Mar 9;10:23743735231160421. doi: 10.1177/23743735231160421 (PMC10009027; doi:10.1177/23743735231160421)
Supplement: sj-pdf-2-jpx-10.1177_23743735231160421 - Supplemental material for Empowering Patients With a Shared Communication Tool: A Patient-Oriented Multimethods Pilot Study [file sj-pdf-2-jpx-10.1177_23743735231160421.pdf]

UNIVERSITY OF ALBERTA
HEALTH SCIENCES COUNCIL
Health Sciences Education and Research Commons

Alberta Health Services
Glenrose Rehabilitation Hospital

JARGON ALERT! / WAIT CARD CLINICIAN USER GUIDE

Why use this card

- Easy way to enhance your communication with patients and families by providing a shared tangible tool
- Patients and families may feel reluctant to verbally interrupt - the card encourages and empowers patients and families to take a more active role in their appointment and care
- Acknowledges patients and families may fear not understanding and feel overwhelmed, especially when the stakes are high

Why the card was created

The **Jargon Alert!** card is a tool to support clear communication.

- People don't always know they are using jargon
- Can provide an easy way to address jargon, unfamiliar language and concepts

The **WAIT** (Why Am I Talking) card can assist in advocating for clear communication in team settings.

- Empowers patients and families to take a moment if they feel overloaded with information

Before using the card

Setting the stage helps all users have a positive experience.

- Explain the purpose of the card to support a mutual understanding
- Ensure all involved are comfortable and welcome use of the card

Example of how to share and use this card

	**JARGON ALERT!**	**WAIT (Why Am I Talking)**
**Before**	*Please use this card to let me know that I need to be more clear about something. It's important that you feel comfortable with the information I share today.*<br><br>*I want to be sure you get what you need from our conversation. You are welcome to use this card if what I am saying is not making sense or you have a question.*	*As we work together today please use this card if you want me to pause. I really want the appointment to be helpful.*<br><br>*During our appointment today if I am speaking too fast or you are feeling overwhelmed, hold up this WAIT card to let me know if I need to slow down or we need a check in.*
**During**	*Please feel free to signal me with the card at any time.*<br><br>**If you get carded...**<br>*Thanks for letting me know something I said isn't clear.*	*It's ok to take a break if you are feeling overloaded.*<br><br>**If you get carded...**<br>*Thank you for letting me know that you need a break. When you're ready, let me know if you have any questions about our conversation so far.*
**After**	*How did you feel using the card?*<br><br>*Did you find the card helpful?*<br><br>*We covered a lot of information. It's ok if you have more questions. Is there anything you want to revisit from our conversation?*	*What was your experience using the card?*<br><br>*Did you find the card helpful?*<br><br>*This is a lot to take in. Is there anything you want to review from our conversation? Do you need any additional support?*

DRAFT

Ways the card can be used

USER 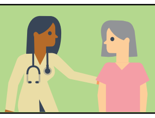	JARGON ALERT!	WAIT (Why Am I Talking)
**Patient and Family Member** Interaction - One to One<br><br>(To be provided to patient for their appointment)	• Informs care provider that they are using medical jargon or acronyms.<br>• Supports patient-friendly plain language.	• Informs care providers that the patients or family have a question about what is being discussed.<br>• Empowers patients or family to pause the discussion due to emotional response or needing a break.
**Patient and Family Member** Interaction - Group Setting<br><br>(To be handed out at workshops and education sessions)	• Informs the facilitator or instructor that medical jargon or acronyms need clarification.	• Informs the facilitator or instructor that the patients or family have a question about what is being discussed.
**Interprofessional interactions among care providers and staff**	• Informs peers and colleagues that medical jargon or acronyms need clarification.<br>• Can be used by new staff during orientation and interprofessional shadowing.	• Can be used as a self-check to remind yourself to speak with purpose and make space for others to contribute. E.g. place the card in your view during meetings.<br>• Can be used by new staff during orientation to signal that they feel overloaded.
**Students**	• Informs peers and mentors that medical jargon or acronyms need clarification.<br>• Can be used in the classroom, small group activities, and interprofessional shadowing.	• Empowers students to pause the discussion due to needing clarification or feeling overloaded.<br>• Can be used in educational and clinical settings.

The Jargon Alert! card is by Alberta Health Services and University of Alberta as part of the Interprofessional Clinical Learning Unit (IPCLU) project.

The source for the "WAIT" acronym is unknown, but is commonly used as a team communication tool in the business sector. The WAIT Card is by the Health Sciences Education and Research Commons (HSERC).
