# Supplementary material for: Empowering Patients With a Shared Communication Tool: A Patient-Oriented Multimethods Pilot Study
Source: J Patient Exp. 2023 Mar 9;10:23743735231160421. doi: 10.1177/23743735231160421 (PMC10009027; doi:10.1177/23743735231160421)
Supplement: sj-pdf-4-jpx-10.1177_23743735231160421 - Supplemental material for Empowering Patients With a Shared Communication Tool: A Patient-Oriented Multimethods Pilot Study [file sj-pdf-4-jpx-10.1177_23743735231160421.pdf]

# I HAVE A QUESTION

UNIVERSITY OF ALBERTA

Alberta Health Services

Glenrose Rehabilitation Hospital

***You can Say...***

Please slow down.

Can you help me understand?

Please repeat what you said.

Can you explain it in a different way?

Can you write it down?

For any questions related to using this card, please contact **sjk1@ualberta.ca**
